# The Effect of N‐Acetylcysteine (NAC) on Neurometabolites and Cognitive Function in Adults With Alcohol Use Disorder: A Preliminary Randomized Controlled Trial

**DOI:** 10.1002/npr2.70066

**Published:** 2025-10-23

**Authors:** Kristiane Yacou Dunbar, Gezelle Dali, Marilena M. DeMayo, Warren Logge, Tristan Hurzeler, Catherine Kelly, Joshua Watt, Lindsay M. Squeglia, Anna E. Kirkland, Paul S. Haber, Kirsten C. Morley

**Affiliations:** ^1^ Specialty of Addiction Medicine, Sydney Medical School, Faculty of Medicine and Health University of Sydney Sydney New South Wales Australia; ^2^ Edith Collins Centre for Translational Research (Alcohol, Drugs & Toxicology) Royal Prince Alfred Hospital, Sydney Local Health District Sydney New South Wales Australia; ^3^ Department of Radiology and Department of Psychiatry University of Calgary Calgary Alberta Canada; ^4^ Department of Psychiatry and Behavioral Sciences Medical University of South Carolina Charleston South Carolina USA

**Keywords:** alcohol use disorder, glutamate, glutathione, magnetic resonance spectroscopy, NAA, N‐acetylcysteine, neurometabolites, pharmacotherapy

## Abstract

**Background and Aims:**

Preclinical studies have demonstrated that N‐acetylcysteine stabilizes levels of glutamate and glutathione and reduces alcohol‐seeking behaviors, indicating it as a potential pharmacotherapy for the management of alcohol use disorder. In this preliminary study, we examined brain metabolite levels and cognitive functioning in individuals with alcohol use disorder enrolled in a randomized controlled trial of N‐acetylcysteine versus placebo.

**Methods:**

In this preliminary trial, 23 participants (average age = 49; 70% male) with moderate to severe alcohol use disorder (DSM‐5) were randomized to receive 2400 mg/day of N‐acetylcysteine (*N* = 9) or placebo (*N* = 14). At baseline and follow‐up (*M* = 19 days; SD = 3.73 days post‐baseline), participants underwent proton magnetic resonance spectroscopy (^1^H‐MRS) to assess levels of glutamate (Glu), glutathione (GSH) and total N‐acetylaspartate (tNAA) in the anterior cingulate cortex (ACC) and completed the Stroop Color and Word Test (SCWT; a measure of distractor interference and cognitive control) and the Trail Making Test (TMT; a measure of set shifting ability).

**Results:**

There were no significant differences between the N‐acetylcysteine or placebo groups in neurometabolite concentrations (GSH/Cr: *p* = 0.75, CI; −0.12–0.09, tNAAG/Cr: *p* = 0.797, CI; −0.10–0.13, Glu/Cr: *p* = 0.60, CI; −0.19–0.32), or cognitive scores (Stroop: *p* = 0.57, CI; −306.93–172.78, TMT: *p* = 0.166, CI; −6.62–36.77).

**Conclusion:**

These preliminary findings indicate that N‐acetylcysteine may not alter brain neurometabolite levels within the ACC or show improvements in certain domains of cognitive functioning measured by the SCWT and TMT, specifically resistance to distractor interference and set‐shifting ability respectively, in individuals with alcohol use disorder.

**Trial Registration:**

ClinicalTrials.gov identifier: NCT03879759

## Abstract 2

First study to examine the effect of NAC on metabolites in adults with alcohol use disorder. No significant differences were observed between NAC and placebo treatment on metabolites or cognitive functioning.

## Graphical abstract

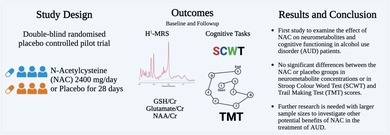

## 1 Introduction

Alcohol use disorder (AUD) is a persistent and recurrent condition characterized by problematic patterns of alcohol consumption with negative biological and behavioral implications that are associated with a considerable disease burden [1]. Within the brain, chronic heavy alcohol use leads to decreased glutamate transporter (GLT‐1) protein levels, disrupting glutamatergic input from the prefrontal cortex to reward areas [2]. This change in excitatory neurotransmission, in addition to the toxic effects of alcohol, can lead to oxidative stress associated with reduced glutathione (GSH; endogenous antioxidant) levels and diminished neural function [3]. AUD has also been associated with dysregulated levels of N‐acetylaspartate (NAA) across a range of brain regions such as the frontal cortex and anterior cingulate cortex [4]. NAA is a marker for neuronal integrity that has been associated with impaired cognitive functioning in AUD [5]. Cognitive deficits can impair the capacity for self‐regulation of alcohol consumption, problem‐solving, and the execution of decisions necessary for successful treatment adherence and long‐term recovery [6, 7]. Treatments that target neurobiological imbalances may prevent further oxidative stress and neuroinflammation, aid in reducing resulting cognitive impairments, and ultimately lessen the symptoms of AUD through regulation of alcohol‐seeking behaviors [8].

While various pharmacotherapies are approved for managing AUD, their overall efficacy in reducing drinking behaviors is modest, and more treatment options are required [9]. N‐acetylcysteine (NAC) is a cysteine precursor that replenishes the intracellular levels of GSH, a key antioxidant synthesized in cells [10]. NAC has emerged as a potential pharmacotherapy for AUD [11] due to its antioxidant properties and potential to restore the balance of GLT‐1 expression and cystine‐glutamate exchange [12, 13]. Pre‐clinical studies have observed NAC to both reduce alcohol‐seeking behaviors [14, 15] and mitigate against increased oxidative stress [16] and also ethanol‐induced oxidative stress (oxidized/reduced glutathione ratio; GSSG/GSH) ([17]).

Clinical trials have found NAC to be well‐tolerated and effective across various neuropsychiatric disorders including substance use [18]. NAC has been found to reduce the severity of cocaine dependence and promote abstinence at dosages of up to 2400 mg/day [19]. It has also been found to decrease daily smoking and alleviate depression in individuals with tobacco use disorder [20], as well as reducing alcohol consumption in individuals with cannabis use disorder [21, 22]. In a recent randomized controlled trial of NAC (2400 mg/day) for the management of AUD, there were no treatment effects over time for heavy drinking days or craving; however, there was a significant interaction between time and treatment, indicating that NAC was more effective at reducing standard drinks per drinking day relative to placebo during the first 7 days of treatment [23]. There have also been mixed effects in terms of the impact of NAC on cognitive functioning in individuals with substance use disorders. One study in individuals with cocaine use disorder observed that NAC improved response inhibition relative to placebo, as measured by the Stop Signal Task. However, NAC did not improve resistance to distractor interference, as measured by classic Stroop performance [19]. Further, one study found no effect of adjunctive NAC treatment relative to placebo on cognition in individuals with bipolar disorder [24], while another study, in individuals with psychosis, reported enhanced working memory performance but no improvement in measures of attention or executive control [25]. There has been no study to date that has examined the effect of NAC on cognitive functioning in adults with AUD.

Studies using proton magnetic resonance spectroscopy (^1^H‐MRS), a non‐invasive technique that can measure neurometabolite levels in human brains, have shown altered neurometabolites in individuals with AUD [4]. The ACC was selected as the region of interest due to its central role in executive function, emotional regulation, and decision‐making—domains commonly impaired in individuals with AUD [26, 27]. Additionally, several ^1^H‐MRS studies have examined neurometabolite profiles in the ACC [28, 29, 30], and multiple studies have focused specifically on the ACC when investigating the effects of NAC [31, 32, 33, 34]. Pharmaco‐MRS studies can examine the potential effect of pharmacological interventions on this neurobiological dysregulation wherein medications found to be effective in reducing alcohol consumption can also modulate neurometabolite levels, such as observed with gabapentin [35] and baclofen [36]. There has been one pharmaco‐MRS study of NAC versus placebo in non‐treatment seeking adolescents with moderate alcohol consumption [37]. This study found no significant effect of treatment on GSH, NAA, or glutamate [37], which may have been due to age and/or shorter durations of use with overall lower levels of consumption compared to treatment‐seeking adults with AUD. No study has yet employed ^1^H‐MRS to examine the effect of NAC on neurometabolites in heavy drinking treatment‐seeking adults with AUD.

In this preliminary substudy of a larger clinical trial [23], we aimed to investigate the possible effect of NAC on neurometabolite levels and cognitive functioning in adults with AUD. The SCWT and TMT were selected due to their sensitivity to cognitive impairments commonly observed in AUD, particularly in the domains of response inhibition and set‐shifting [38, 39]. Although previous studies have reported no effect of NAC on these specific tasks [19, 24], they remain well‐validated and widely used measures in substance use research. We acknowledge that these two tasks only capture select aspects of cognitive functioning and do not represent the full spectrum of cognitive domains potentially affected in AUD. We hypothesized that treatment with NAC (2400 mg/day) versus placebo will lead to: (i) increases in GSH, total NAA (tNAA), and decreased glutamate levels in the anterior cingulate cortex (ACC) as measured by ^1^H‐MRS, and (ii) improved performance on the Stroop Color Word Test (SCWT), as indicated by a reduced interference effect (i.e., reduced difference in latency times between incongruent and control conditions), and improved set‐shifting performance on the Trail Making Test (TMT), as indicated by a reduced difference in completion time between Part A and B (i.e., Part B–Part A).

## 2 Materials and Methods

This study was approved by the Human Ethics Review Committee of the Sydney Local Health District (X17‐0343 & 2019/STE08617). All participants included in this ^1^H‐MRS sub‐study provided written informed consent after the commencement of randomization for the main trial.

### 2.1 Participants

Participants were recruited from a randomized, double‐blind, placebo‐controlled trial of N‐acetylcystine versus placebo in the treatment of AUD. Details and results for the main study have been reported elsewhere [23]. Briefly, all participants were recruited from the Royal Prince Alfred Hospital inpatient detoxification program, outpatient treatment or follow‐up, or who had responded to advertising. Eligible participants were classified according to the DSM‐5 criteria for AUD. Inclusion criteria included: (i) the desire to reduce or completely stop drinking; (ii) consumed at least 21 standard drinks per week or had 2 heavy drinking days per week (HDD: ≥ 5 standard drinks/day for men; ≥ 4 for women) within 30 days prior to screening; (iii) aged between 18 and 70 years; (iv) possessing adequate cognition and English language skills for valid consent and research interviews; and (v) willingness to provide written informed consent. Exclusion criteria included: (i) pregnancy or lactation; (ii) concurrent usage of any psychotropic medication apart from stable‐dose antidepressants for at least 2 months; (iii) substance use disorder other than nicotine; (iv) unstable medical conditions (e.g., cancer) or psychiatric disorders (e.g., active psychosis, schizophrenia, or elevated suicidal risk hindering trial participation); (v) lack of stable housing; (vi) simultaneous use of selenium, vitamin D, or other antioxidants; and (vii) any alcohol pharmacotherapy within the preceding month; (viii) presence of any metal implants or foreign bodies; (ix) claustrophobia. From the 42 total participants in the main trial, 23 participants consented to the ^1^H‐MRS sub‐study including 14 allocated to the placebo arm and 9 allocated to NAC.

### 2.2 Procedure

Participants completed two ^1^H‐MRS scanning sessions at baseline (T0) and at follow‐up (T1) (*M* = 19 days; SD = 3.73 days). Participants were randomized into one of two medication groups (NAC 2400/day and placebo) in a 1:1 ratio. The random allocation, generated by a computer‐based block randomization process, was kept confidential and handled by an independent Clinical Trials Pharmacist responsible for medication dispensing. All parties involved, including researchers, clinicians, and patients, were unaware of the treatment assignments to ensure the study's double‐blind integrity. Medications in both groups were also encapsulated to maintain blinding throughout the study. In the follow‐up scan, participants were scanned approximately 120 min post administration of either NAC or placebo. Breath alcohol concentration was obtained, and only participants with a reading of 0.00 g% were permitted to proceed with the scan.

### 2.3 Assessments

A detailed list of assessments has been outlined previously [23]. At baseline, comprehensive structured diagnostic information about AUD and participant demographics was gathered. Baseline and follow‐up measures included the following assessments: Timeline Follow Back (TLFB) forms were utilized [40] to assess alcohol consumption over the past month to calculate alcohol consumption variables including standard drinks per drinking day (SDDD) and heavy drinking days per week (HDD/week); the severity of alcohol dependence was assessed using the Alcohol Dependence Scale (ADS) with higher scores indicating greater severity of AUD [41]; alcohol craving was measured using the Penn Alcohol Craving Scale (PACS) [42]; levels of depression, anxiety, and stress were measured using the Depression Anxiety Stress Scale (DASS‐42) [43]; and sleep issues were determined using the Insomnia Severity Index (ISI) [44].

#### 2.3.1 Stroop Color Word Test (SCWT)

The SCWT [45] is a decision‐making measure used to assess resistance to distractor interference [46]. Participants are presented with color words in incongruent (e.g., “red” printed in green), congruent (e.g., “red” in red), or control conditions (e.g., non‐linguistic symbols in color). In all trials, participants are instructed to name the ink color while ignoring the words' semantic meaning. The task was administered in a computerized format based on the Golden version [47] with 24 stimuli per condition and no time limits. Individual response latencies were recorded for each trial. The Stroop interference score was calculated as the difference in mean response time between incongruent and control conditions (incongruent—control). This method was selected as control trials provide a neutral baseline without semantic facilitation, offering a more precise estimate of interference. Although the congruent condition was included for comparison, it was not used in the interference score. Although the congruent condition was not used in calculating the interference score, its inclusion alongside incongruent trials enhances the SCWT's sensitivity in detecting cognitive impairments [48]. This rationale aligns with the original Stroop paradigm, where interference was defined as the delay in color‐naming for incongruent versus neutral stimuli [45], and is supported by recommendations to use control trials to isolate interference effects [49, 50]. Higher interference scores reflect poorer executive functioning [48] resulting from greater Stroop interference.

#### 2.3.2 Trail Making Test (TMT)

The TMT, introduced in 1938 by Partington and Leiter [51], is a broader assessment of executive functioning. It consists of two parts: Part A requires participants to connect numbered circles in ascending order and is a primary measure of attention. Part B involves alternating between numbers and letters in consecutive order (e.g., 1‐A‐2‐B‐3‐C) and assesses both attention and set‐shifting ability. The difference in time taken to complete Part A and B (i.e., Part B–Part A) is used to assess cognitive flexibility and set‐switching abilities, and a greater difference score indicates poor set‐shifting. The TMT provides valuable insights into set‐shifting flexibility, attention, and inhibition—essential components of executive functioning [52].

### 2.4 ^1^H‐MRS

MRS data was collected on a 3T GE Discovery 750 Scanner. A T1‐weighted anatomical scan (TR = 7.2 ms; TE = 2.8 ms; flip angle = 10°; matrix 256 × 256; 0.9 mm isotropic voxels, 196 slices) was collected for voxel placement and co‐registration. A single voxel PRESS ^1^H‐MRS acquisition was acquired (128 water suppressed and 24 unsuppressed averages, TR: 2000 ms, TE: 35 ms, 4096 points, bandwidth 5000 HZ). The voxel (voxel size: 20 × 20 × 20 mm) was placed in the anterior cingulate cortex (ACC) and shimmed to achieve line widths (FWHM) of < 15 Hz. Data were pre‐processed using the automated FID‐A pipeline, which includes coil combination, removal of bad averages (CRLB < 20% for each metabolite followed by visual inspection), and spectral registration [53], and metabolites were quantified using LCModel [54]. The basis set for quantification was based on simulations using the FID‐A toolbox [53] with sequence specific timing and radiofrequency pulse shape. The basis set included: alanine, aspartate, glycerophosphocholine, phosphocholine, creatine, phosphocreatine, gamma‐aminobutyric acid (GABA), glutamate, glutamine, lactate, inositol, NAA, total N‐acetyl aspartate (tNAA), scyllo‐inositol, glutathione, glucose, and taurine, with the default macromolecule simulation parameters from LCModel. Three GSH data points were flagged for quality control based on CRLB < 20%; however, they were retained following visual inspection to ensure good fit. The metabolites of interest were investigated using metabolite referenced to both creatine (Cr) to account for differences in water signal between time points and water, and the two datasets were checked for consistency. Voxel co‐registration was performed using Gannet [55], and similar voxel composition (GM, WM, and CSF) between each time point was confirmed.

### 2.5 Statistical Analysis

Differences between groups on baseline variables were determined using a two‐sample test of independence for continuous variables and a chi‐squared test of independence for categorical variables. Linear mixed models were used to examine medication differences (NAC vs. placebo) for the neurometabolites (Glu/Cr, GSH/Cr, tNAA/Cr) and cognitive tests (SCWT and TMT), with time and treatment and time × treatment as fixed effects and participant as random effects. Water referenced neurometabolites were also examined to check for consistency. Changes in cognitive functioning were specifically measured as performance on the Stroop test—indicated by the difference in incongruent and control latency times—and the TMT—indicated by the difference in completion time between Parts A and B (Part B–Part A). Antidepressant use, ISI, DASS stress, and recent drinking (T0 and T1) were included as predefined covariates in the analysis to be consistent both with our main pilot trial [23] and fMRI analyses [56]. The “Recent Drinking” covariate refers to whether a participant consumed alcohol within 24 h prior to each ^1^H‐MRS scan. This was recorded as a binary variable (“1 = yes” / “0 = no”) based on participant self‐report immediately prior to scanning. All analyses were two‐tailed, with an alpha level of 0.05. All fixed‐effect estimates reported are unstandardized beta coefficients, presented in the original metric of the outcome (e.g., seconds for TMT), along with corresponding 95% confidence intervals and *p*‐values. Data were analyzed using the lmer () function from the lme4 package in R version 4.3.2 (2023‐10‐31).

## 3 Results

### 3.1 Sample Characteristics

**FIGURE 1:**
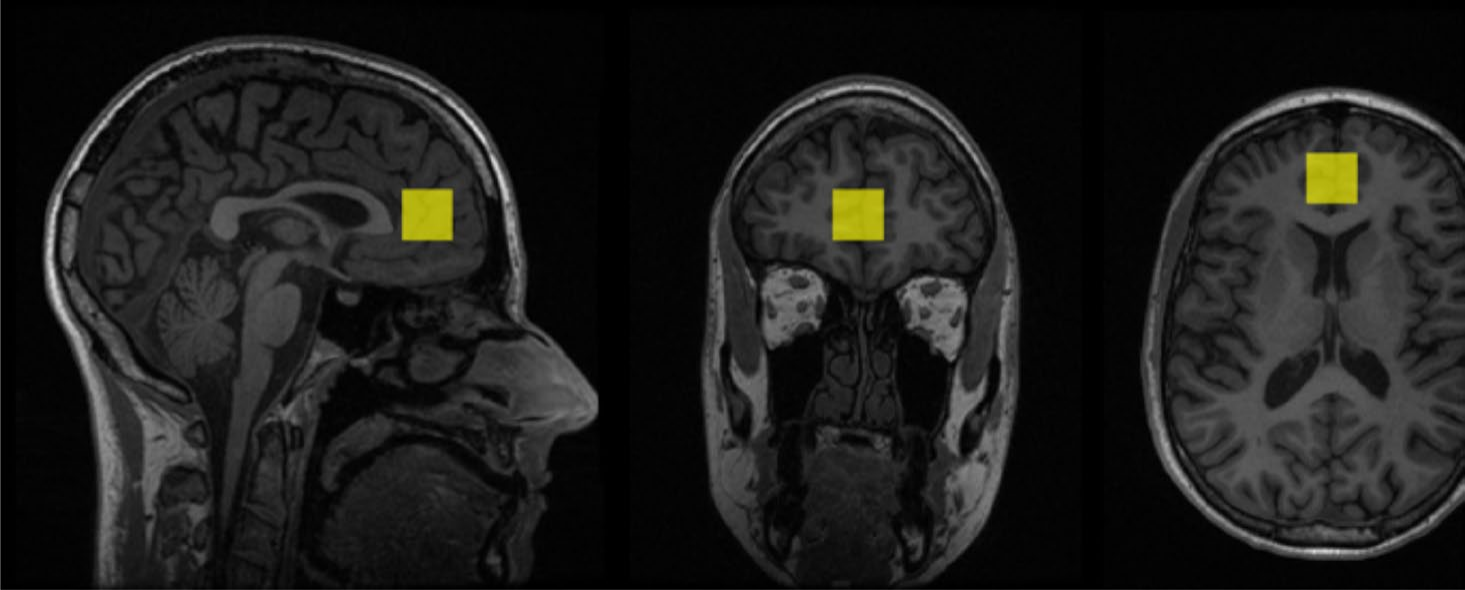
T1‐weighted structural image showing the anatomical localization of acquisition voxel.

From the 42 total participants in the main trial, 23 participants consented to the ^1^H‐MRS sub‐study, including 14 allocated to the placebo arm and 9 allocated to NAC (Figure 1 depicts the flow of participants through the MRS sub‐study). Baseline characteristics are presented in Table 1. Overall, the participants were approximately 49 years (±11.75), 70% male, and consumed an average of approximately 14.98 SDDD (±0.895) at the time of enrolment. Additionally, 47% were concurrently taking anti‐depressant medications. Alcohol consumption within 24 h prior to scanning was reported at 41% at baseline and 48% at follow‐up, but 100% demonstrated a breath alcohol concentration of 0.00 g% 120 min prior to scanning. Both groups demonstrated similar levels of SDDD prior to treatment, as well as comparable proportions of alcohol‐related liver disease and antidepressant use (*p*'s > 0.214). There were no significant group differences with regard to alcohol severity as measured by ADS (*p* > 0.4) and no significant group differences in DASS scores (*p*'s > 0.169) or severity of insomnia measured by ISI (*p* > 0.264).

**TABLE 1:** Baseline characteristics.

Characteristics	Placebo (*n* = 14)	NAC (*n* = 9)	*p*	Effect size
Age, y	48.1 ± 11.57	51.7 ± 12.72	0.503	Hedges' *g* = 0.29
Gender, %F	21.0%	44.0%	0.480	Cramér's *V* = 0.15
Baseline SDDD^a^	16.96 ± 7.68	11.97 ± 7.68	0.214	Hedges' *g* = −0.57
Baseline HDD/week^b^, ^c^	3.1 ± 2.95	2.7 ± 2.87	0.994	Hedges' *g* = 0
ADS score^d^	20.5 ± 10.34	24.22 ± 9.53	0.400	Hedges' *g* = 0.35
Alcohol‐related liver disease^e^, %	35.7	22.2	0.824	Cramér's *V* = 0.05
Antidepressants, %	40.0	55.5	0.827	Cramér's *V* = 0.5
ISI	9.92 ± 6.11	13 ± 5.51	0.264	Hedges' *g* = 0.49
DASS stress	15.14 ± 10.88	20.89 ± 7.95	0.169	Hedges' *g* = 0.55
DASS anxiety	11.86 ± 9.44	11.33 ± 4.12	0.860	Hedges' *g* = −0.06
DASS depression	14.57 ± 11.71	12.00 ± 7.00	0.527	Hedges' *g* = −0.24

*Note:* Data represent *M* ± SD unless otherwise noted. *p*‐values from independent *t*‐tests (continuous variables) or chi‐square tests (categorical variables). Hedges' *g* used for effect sizes in continuous variables; Cramér's V for categorical variables.

Abbreviations: ADS, Alcohol Dependence Severity Scale; DASS, Depression Anxiety Stress Scale; ISI, Insomnia Severity Index; NAC, *N*‐acetylcysteine.

^a^ Defined as standard drinks per drinking day.

^b^ Calculated from the 28 days preceding the first day of the study, based on the Time‐Line Follow‐Back method.

^c^ Defined as ≥ 4 drinks for women and ≥ 5 drinks for men.

^d^ As measured by Alcohol Dependence Severity scale (ADS).

^e^ Defined as the presence of symptoms and/or signs referable to liver disease or its complications with or without cirrhosis, in which alcohol use is considered to play a major aetiological role.

### 3.2 Metabolite Concentrations Following Treatment With NAC Versus Placebo

**FIGURE 2:**
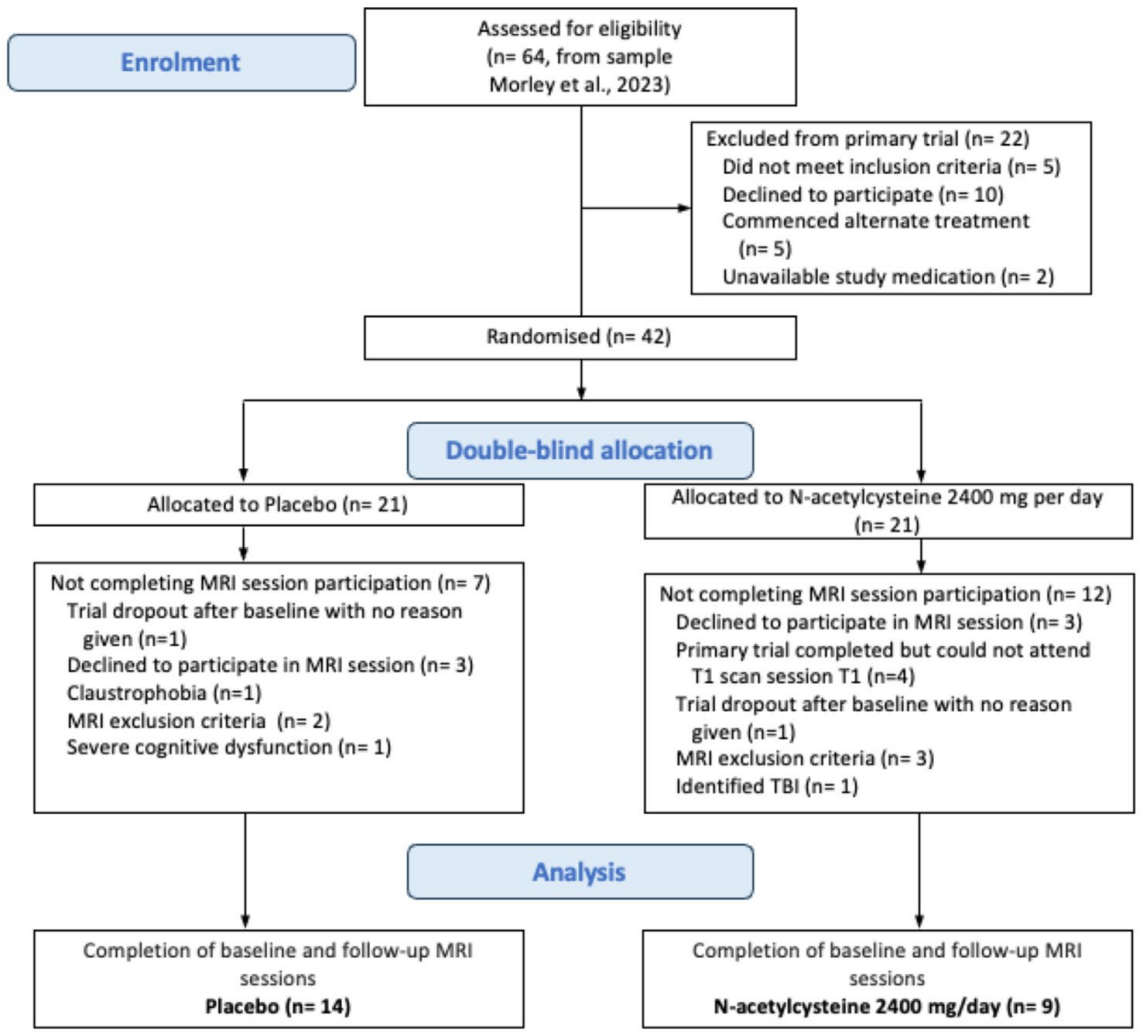
Flow of participants through the study.

**FIGURE 3:**
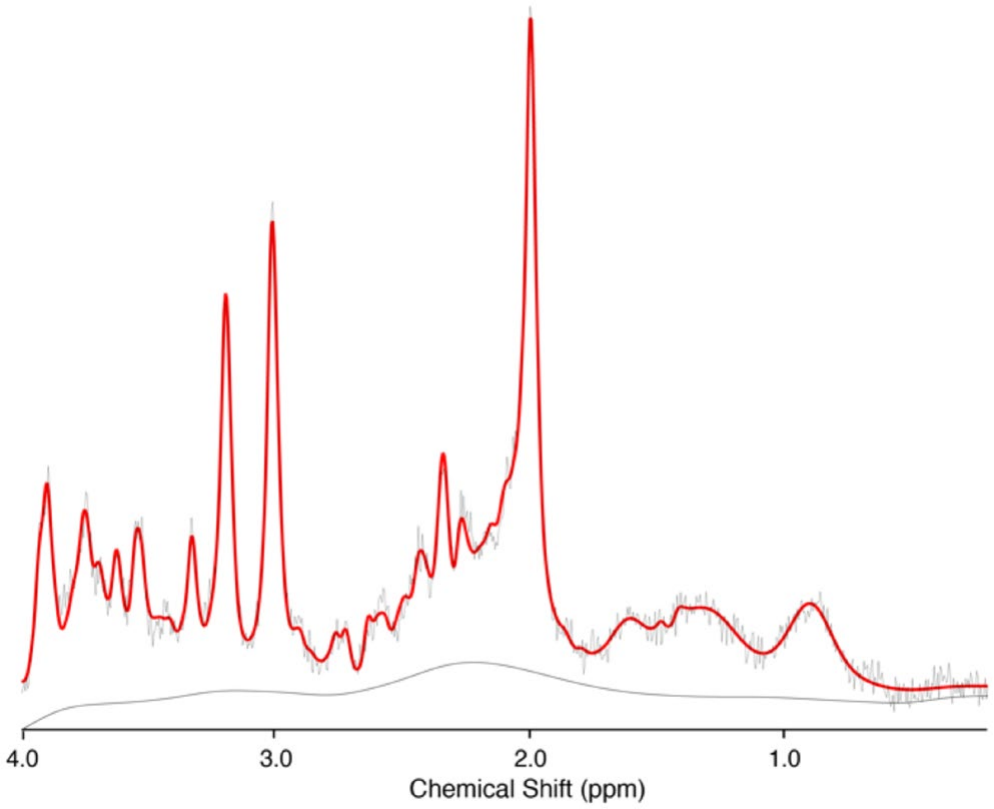
Example MR‐spectrum from the ACC.

Figures 2 and 3 represent example voxel placement and spectrum from the ACC respectively. Table 2 presents the baseline and follow‐up mean concentrations of the three neurometabolites (GSH, tNAA, and Glutamate). Mixed models revealed there were no significant differences between groups (NAC vs. placebo) on metabolite concentrations (*p*'s > 0.45) or for time (*p*'s > 0.30) or any time × treatment effects (GSH/Cr: *p* = 0.35, CI; −0.14–0.05, tNAA/Cr: *p* = 0.77, CI; −0.12–0.09, Glu/Cr: *p* = 0.61, CI; −0.22–0.37; see Table 3). The results for creatine and water referenced values were consistent across all analyses.

**TABLE 2:** Baseline and follow‐up mean (+/− SD) ^1^H‐MRS concentrations of neurometabolites creatine corrected and SCWT and TMT scores in placebo and NAC‐treated groups.

Neurometabolite/Cr	Placebo (*n* = 14)		NAC (*n* = 9)	
	Baseline	Follow up	Baseline	Follow up
GSH/Cr	0.20 ± 0.03	0.20 ± 0.08	0.21 ± 0.08	0.17 ± 0.04
tNAA/Cr	1.24 ± 0.14	1.21 ± 0.15	1.21 ± 0.11	1.15 ± 0.08
Glutamate/Cr	1.39 ± 0.26	1.41 ± 0.14	1.41 ± 0.14	1.41 ± 0.10
Cognitive measure				
SCWT (interference score, sec)	218.84 ± 223.27	224.22 ± 282.64	239.29 ± 233.84	208.07 ± 77.92
TMT (Part B–A, sec)	43.48 ± 21.03	39.70 ± 20.07	40.83 ± 18.10	43.04 ± 20.34

*Note:* Values represented are means±SD.

Abbreviations: Cr, creatine corrected; GSH, glutathione; NAC, *N*‐acetylcysteine; SCWT, Stroop Color Word Test: values represent interference score (seconds), calculated by subtracting the mean response latencies of control trials from those of incongruent trials; TMT, Trail Making Test: values represent the difference in time (seconds) between Part B and Part A.; tNAA, total N‐acetyl aspartate.

**TABLE 3:** Linear mixed effect models for neurometabolites GSH, tNAA, and Glutamate.

Predictors	GSH/Cr			tNAA/Cr			Glutamate/Cr		
	Estimates	CI	*p*	Estimates	CI	*p*	Estimates	CI	*p*
(Intercept)	0.20	0.12–0.28	**< 0.001**	1.28	1.17–1.40	**< 0.001**	1.53	1.30–1.75	**< 0.001**
Timepoint [FU]	0.02	−0.04–0.09	0.462	−0.04	−0.12–0.04	0.298	−0.06	−0.27–0.15	0.54
Treatment group	0.00	−0.08–0.08	0.919	−0.04	−0.15–0.07	0.451	−0.03	−0.26–0.20	0.786
Antidepressants	0.01	−0.05–0.07	0.782	0.02	−0.06–0.11	0.586	−0.07	−0.23–0.09	0.382
Recent drinking	−0.02	−0.08–0.03	0.432	−0.02	−0.09–0.05	0.533	0.01	−0.14–0.17	0.851
ISI	0.00	−0.01–0.00	0.522	0.00	−0.01–0.00	0.218	−0.01	−0.02–0.01	0.341
DASS stress^a^	0.00	−0.00–0.00	0.608	0.00	−0.00–0.01	0.814	0.00	−0.01–0.01	0.829
Timepoint [FU] × treatment group^a^	−0.04	−0.14–0.05	0.352	−0.02	−0.12–0.09	0.773	0.07	−0.22–0.37	0.613
Random effects									
*σ* ^2^	0.00			0.04			0.01		
τ_00 PID_	0.00			0.00			0.00		
ICC	0.23			0.04			0.41		
*N*_PID_	16			16			16		
Observations	32			32			32		
Marginal *R* ^2^/conditional *R* ^2^	0.079/0.292			0.081/0.113			0.201/0.528		

*Note:* Bold represent significant *p* values.

Abbreviations: DASS, depression and anxiety stress scale; ICC, intra‐class correlation coefficient; ISI, insomnia severity index; *σ* ^2^, random effects variance; τ00, random intercept variance.

^a^ Timepoint [FU] × treatment group = interaction term reflecting the effect of NAC versus placebo over time.

### 3.3 Executive Function Tasks Following Treatment With NAC Versus Placebo

Table 2 presents the baseline and follow‐up values for SCWT and TMT. Mixed models (depicted in Table 4) revealed no significant differences for SCWT over time (*p* = 0.69, CI; −204.75–137.56), treatment (PL vs. NAC; *p* = 0.99, CI; −320.55–323.18), or time × treatment (*p* = 0.89, CI; −266.04–231.29), nor for TMT over time (*p* = 0.11, CI; −30.42–3.37), treatment (*p* = 0.29, CI; −31.56–9.82), or time × treatment (*p* = 0.20, CI; −9.09–41.03).

**TABLE 4:** Linear mixed effects models for cognitive functioning.

Predictors	SCWT			TMT		
	Estimates (*β*)	CI	*p*	Estimates (*β*)	CI	*p*
(Intercept)	257.91	−78.10–593.92	0.126	45.78	25.79–65.77	**< 0.001**
Timepoint [FU]	−33.6	−204.75–137.56	0.689	−13.52	−30.42–3.37	0.111
Treatment group	1.32	−320.55–323.18	0.993	−10.87	−31.56–9.82	0.288
Antidepressants	−25.18	−303.89–253.52	0.854	17.24	1.73–32.74	**0.031**
Recent drinking	8.25	−164.99–181.50	0.922	0.29	−14.33–14.92	0.967
ISI	−1.57	−26.48–23.34	0.898	−1.13	−2.49–0.24	0.102
DASS stress	1.94	−12.97–16.85	0.79	0.54	−0.29–1.37	0.19
Timepoint [FU] × treatment group^a^	−17.37	−266.04–231.29	0.887	15.97	−9.09–41.03	0.200
Random effects						
*σ* ^2^	30556.53			297.51		
τ_00 PID_	52007.49			52.96		
ICC	0.63			0.15		
*N*_PID_	17			17		
Observations	34			33		
Marginal *R* ^2^/conditional *R* ^2^	0.011/0.634			0.287/0.394		

*Note:* All fixed‐effect estimates are unstandardized beta coefficients (*β*) presented in original outcome units (seconds for SCWT and TMT). Bold represents significant *p* values.

Abbreviations: DASS, depression and anxiety stress scale; ICC, intra‐class correlation coefficient; ISI, insomnia severity index; *σ* ^2^, random effects variance; τ00, random intercept variance.

^a^ Timepoint [FU] × treatment group = interaction term reflecting the effect of NAC versus placebo over time.

## 4 Discussion

This preliminary neuroimaging substudy from a larger clinical trial examined the effect of NAC treatment versus placebo on neurometabolite concentrations and cognitive functioning. It was expected that treatment would lead to elevations in GSH, tNAA, and decreases in glutamate concentrations within the ACC, alongside improved cognitive functioning, as assessed by the executive function tasks SCWT and TMT. Contrary to our hypotheses, no significant differences in neurometabolite concentrations or cognitive functioning were observed between the groups following treatment, although our results may be limited by a lack of power to detect an effect.

The current results are consistent with previous findings in adolescents with heavy alcohol consumption, which did not observe any effect of NAC (2400 mg/day) on glutamate + glutamine (Glx) or GSH [37]. One previous study in individuals with tobacco use disorder also observed no effect of NAC (2400 mg/day) on glutamate levels within the ACC [57]. Our results are, however, inconsistent with Schmaal, Veltman, Nederveen, van den Brink, and Goudriaan [33]; Schmaal, Veltman, Nederveen, van den Brink, and Goudriaan [33], who found that an acute dose of NAC (2400 mg given 60 min before scan) normalized elevated glutamate levels in the ACC in a small sample of male individuals dependent on cocaine (*n* = 8). It is possible that cocaine dependence is associated with a greater degree of glutamatergic imbalance [58, 59], which would therefore provide a greater window for restoration with NAC treatment. Comparative neuroimaging and preclinical studies indicate that while alcohol, nicotine, and cocaine all involve glutamatergic dysregulation, tobacco use disorder and AUD do not exhibit as significant a glutamate imbalance as cocaine use disorder [60], as cocaine elicits more pronounced changes in glutamate transporters [61]. Inclusion of a healthy control group in future AUD pharmaco‐MRS studies could enable a thorough examination of the role of baseline glutamatergic dysregulation on the effect of medication.

In light of this, the alcohol consumption findings from our pilot study [23] reported a significant difference between NAC and placebo on drinks per drinking day, with the main difference observed at Week 1. The early days of abstinence following cessation of alcohol consumption are marked by the greatest neurometabolite disturbances [29, 30], which could be the optimal therapeutic window for NAC. Thus, the follow‐up scanning time in the current study (on average 19 days into treatment) did not adequately capture this observed therapeutic period. It is also possible that NAC may still act on neurobiological processes that are not adequately captured with the neurometabolites measured with ^1^H‐MRS [37].

Our results also revealed no significant differences between NAC and placebo on performance in the SCWT and TMT. These SCWT results are consistent with previous studies that did not report any significant improvements in the SCWT following NAC (2000–2400 mg/day) versus placebo treatment in individuals with cocaine use disorder over 25 days [19] and in individuals with psychosis over 24 weeks [25]. However, both these studies did observe positive effects of NAC treatment relative to placebo in different cognitive domains such as response inhibition [19] and working memory [25]. Thus, while our results indicate that NAC does not improve executive functioning—specifically, resistance to distractor interference and cognitive control as measured by the SCWT and set shifting ability as measured by the TMT—in heavy drinking individuals with AUD, examination of the effect of NAC in other cognitive domains may be warranted.

Although the time × treatment interaction for the TMT was not statistically significant (*β* = 15.97, *p* = 0.200), the direction of the effect suggests that the NAC group exhibited a relative slowing in TMT performance over time, compared to a slight improvement in the placebo group. This effect may still hold clinical relevance and warrants further investigation in larger, more adequately powered samples. Additionally, a significant main effect of antidepressant use on TMT performance was observed (*β* = 17.24, *p* = 0.031), suggesting that comorbid conditions such as depression or anxiety may influence cognitive performance outcomes in this population. Depression has been consistently associated with impairments in executive functioning, including reduced processing speed, set‐shifting, and cognitive flexibility—domains measured by the TMT [62, 63]. However, there is no consistent evidence that selective serotonin reuptake inhibitors (SSRIs) negatively impact executive function. For example, in a large, randomized study, Shilyansky et al. [64] found no evidence that SSRIs impaired executive function across multiple cognitive domains. Wagner et al. [65] also reported that executive functions such as cognitive flexibility and verbal fluency, as measured by the TMT, improved over the course of antidepressant treatment, and that improvements in these domains tracked with reductions in depression severity. Therefore, the observed association between antidepressant use and poorer TMT performance is more likely due to underlying psychiatric conditions rather than a direct pharmacological effect of the medications. This underscores the importance of accounting for psychiatric comorbidities in future trials assessing cognitive outcomes in AUD.

This study possesses several strengths, including its randomized, double‐blind, placebo‐controlled design with pre and post treatment measurements, thereby enhancing the internal validity of the current research and minimizing potential biases in treatment allocation and assessment. Moreover, recruitment of heavy drinking treatment‐seeking individuals with AUD enhances generalizability to the target clinical population. A notable limitation, however, is the study's small sample size with uneven samples in the NAC and placebo groups at the end of the study, which may have limited our power to detect an effect of NAC on neurometabolite concentrations. Additionally, while ^1^H‐MRS provides detailed metabolic information, it is constrained by its spatial resolution and sensitivity to environmental factors, which might limit the ability to properly measure the dynamic and complex neurometabolites of interest. Further, for feasibility reasons, our cognitive and neurometabolite assessment time point occurred approximately 1 week before the end of treatment visit on the main trial. While this timing was selected to detect early treatment effects, it is possible that cognitive improvements may require a longer intervention period to emerge. However, there were no significant reductions in alcohol consumption at the end of treatment visit in the main trial [23], such that it is unlikely that cognition and neurometabolite concentrations would have been detected with an extended assessment period. Conversely, however, the assessment time point may not have captured potential changes in neurometabolites earlier in the treatment period, in which there was an observed reduction in alcohol consumption in the main trial [23]. Another limitation is that the current study focused exclusively on the ACC, which may have overlooked other potential NAC‐related effects in other brain regions. Future research should consider inducing additional ROIs to provide a more comprehensive understanding of NAC's neurobiological effects. Finally, as described above, the current study only assessed two executive functioning tasks. Further research could examine the effect of NAC on response inhibition and working memory in AUD individuals.

In conclusion, this small substudy is the first study to examine the effect of NAC on neurometabolites and cognitive functioning in this population. While our preliminary findings do not support the hypothesis that NAC treatment modulates levels of neurometabolites or certain domains of cognitive functioning in treatment‐seeking individuals with AUD, these results may be limited by the small sample size.

## Author Contributions

The contributions of the authors are as follows: K.Y.D. contributed to analytic design and methodology, conducted the data cleaning, analysis, and wrote the manuscript. G.D. contributed to analytic design, methodology, and supervised the analytic design. M.M.D. contributed to the design and analysis of MRS. W.L. and T.H. conducted the scans. K.C.M. developed the study concept and design, contributed to manuscript writing, and supervised research staff. P.S.H. contributed to the study concept and design and provided clinical oversight. J.W. was the site physician. All authors contributed to manuscript editing, provided guidance, and approved the final version for submission.

## Ethics Statement

This study was approved by the Human Ethics Review Committee of the Sydney Local Health District (X17‐0343 and 2019/STE08617).

## Consent

All participants included in this ^1^H‐MRS sub‐study provided written informed consent after the commencement of randomization for the main trial.

## Conflicts of Interest

The authors declare no conflicts of interest.

## Supporting information


**Data S1:** npr270066‐sup‐0001‐DataS1.csv.

## Data Availability

De‐identified data is available in the Supporting Information.
